# Assessing sex assignment concordance with genotype and phenotype

**DOI:** 10.1186/1687-9856-2013-7

**Published:** 2013-03-14

**Authors:** Deepa Suresh, Jessica Crawford, Marni E Axelrad, Sheila K Gunn, Laurence McCullough, O’ Brian Smith, Vernon R Sutton, David Roth, Lefkothea P Karaviti, Jennifer E Dietrich

**Affiliations:** 1 Department of Pediatrics, Gender Medicine Team, Baylor College of Medicine, Houston, TX, 77030, USA; 2 Department of OBGYN, Division of Pediatric and Adolescent Gynecology, Baylor College of Medicine, Houston, TX, 77030, USA; 3 Department of Urology, Division of Pediatric Urology, Baylor College of Medicine, Houston, TX, 77030, USA

## Abstract

**Objectives:**

To catalogue patients with DSD and to assess the concordance of genotype and phenotype with sex assignment at birth compared to sex assignment before and following assessment by a Gender Medicine Team (GMT) at one institution, as an initial step in formulating standardized guidelines for management of these conditions.

**Design:**

After obtaining IRB approval, a retrospective chart review was conducted patients seen in the Gender Medicine Clinic (GMC) between 2006–2009 at Texas Children’s Hospital (TCH), Houston, Texas. McNemar’s test and Kappa agreement provided associations of various factors with sex assignment at birth prior to GMT assessment and after GMT assessment.

**Participants:**

Forty-seven patients seen in the GMC with confirmed DSD.

**Results:**

Forty-seven patients met the inclusion criteria. The mean age of the patients at the time of GMT evaluation was 9.1+/−6.1 years; 61.7% had male karyotype, and 38.3% had female karyotype; 51.1% had a male external phenotype, 42.6% had a female external phenotype, and 6.4% had phenotypic ambiguity. Sex assignment was concordant with genotype and phenotype in 63.8% and 86.4%, respectively of cases at the time of birth and in 76.6% and 97.7%, respectively, of cases after assessment by GMT.

**Conclusion:**

Long-term outcomes are needed to establish standardized practice guidelines for decision-making.

## Introduction

Each year, approximately 1 in 3,000 infants is born with a disorder of sexual differentiation (DSD). One of the many challenges, and often a stressful one, particularly for parents, is determining the sex assignment [1-4]. Because of the paucity of studies on outcomes and decision-making criteria that lead to satisfactory sex assignment, practice guidelines for sex assignment have not yet been established. Several clinical guidelines by our group [4] and others [5] have been suggested. However, no previous studies have explored the concordance of gender assignment to genotype and/or phenotype as part of establishing standardized guidelines. Our study goal was to retrospectively assess patients with DSD in order to assess the correlation of genotype and phenotype with sex assignment both before and after assessment by our Gender Medicine Team (GMT).

## Methods

**Figure 1:**
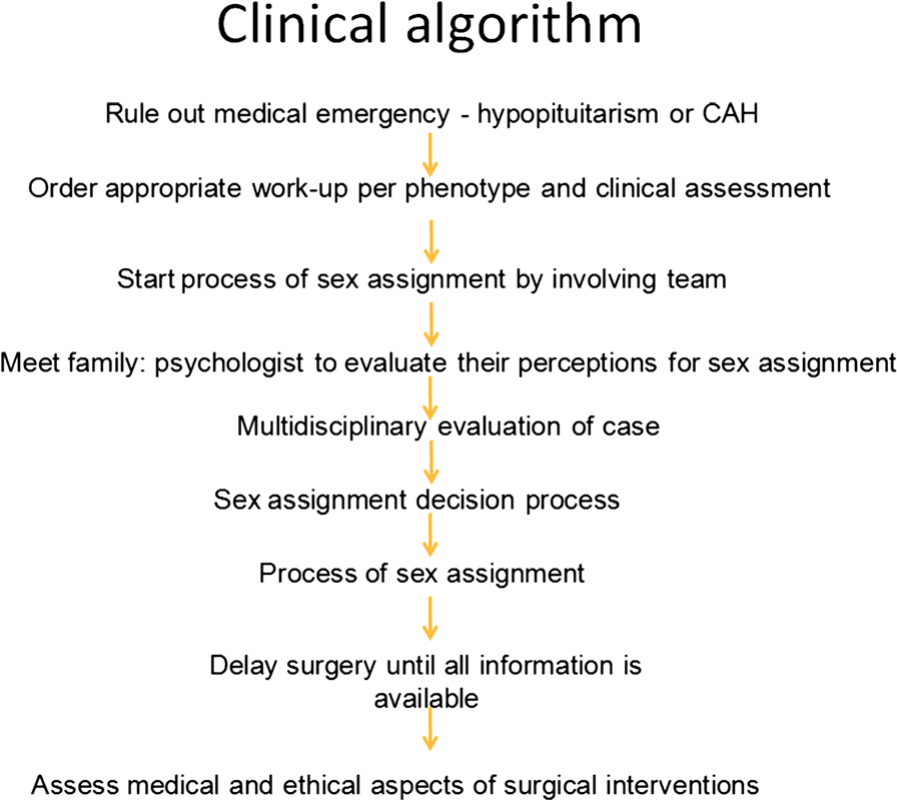
Clinical algorithm for assessment of patients with DSD.

After obtaining IRB approval from Baylor College of Medicine, Houston, Texas, we performed a retrospective chart review of all patients treated in the Gender Medicine Center (GMC) at Texas Children’s Hospital (TCH) in Houston, Texas, between February 2006 and September 2009. The GMC is made up of Pediatric Endocrinology, Pediatric Genetics, Pediatric Urology, Pediatric Gynecology, Social Work, Psychology and Nursing. Inclusion criteria included that patients were seen in the GMC at TCH and had a confirmed DSD. This period preceded the use of an established, ethically based guideline in our Center [6]. We reviewed the charts of 47 patients with DSD treated in the GMC [4], with attention given specifically to history, physical examination [7], hormonal tests, imaging studies (ultrasound primarily, however, MRI was available in some cases), genotype, and final sex assignment. In assessing the genotype, the presence of a Y chromosome or partial Y component was labeled as male genotype [8,9]. A decision tree was used in the assessment and management of all subjects with DSD. It included not only steps for ruling out emergent endocrinopathies, but also specific detailed clinical, laboratory, and karyotype assessments. Furthermore, any interventions needed or sex assignment discrepancies were further assessed to make final determinations about sex assignment after the GMT evaluation was completed (Figure 1). Patients were scheduled to be seen in the GMC as soon as their Pedaitrician made a referral so the time from evaluation at birth to evaluation in GMC was variable. McNemar’s test and Kappa agreement provided associations of various factors with sex assignment at birth and after the GMT’s assessment.

## Results

A total of 47 patients with a variety of underlying diagnostic concerns were enrolled. The most common diagnosis was mixed gonadal dysgenesis (19.2%), followed by hypospadias (19.1%) and congenital adrenal hyperplasia (17%) (Table 1). The mean age of the patients in the cohort was 8.6 yrs (SD +/− 6.14); 61.7% had male karyotype, and 38.3% had female karyotype. Mixed gonadal dysgenesis was determined on the basis of phenotype and karyotype. Of the 47 patients, 51.1% had external male phenotype, 42.6% had external female phenotype, and 6.4% had ambiguous phenotype. Additional characteristics about phenotype were obtained from imaging studies. Evidence of male internal organ anatomy was present in 30.4%. Evidence of female internal organ anatomy was present in 30.4% of cases. Male and female structures were evident in 37% of cases, while no identifiable male or female structures occurred in 2.2% of cases. At birth, 23.4% reportedly had normal male hormonal patterns, and 76.6% had normal female hormonal patterns demonstrated with biochemical testing. Further assessment and biochemical testing by the GMT revealed that 46.8% had normal androgen (17 hydroxy progesterone, dihydroepiandrostenedione, testosterone, dihydrotestosterone) patterns, 10.6% had no evidence of abnormal androgens, and 42.6% had an intermediate pattern of androgen secretion.

**Table 1:** Diagnostic features of cohort

**Diagnosis**	**Age range (years)**	**N = 47 (%)**
Hypospadius	1-7.75	9 (19.1)
Micropenis	4.5-5.75	4 (7.5)
MGD	0.5-25.75	9 (19.2)
VACTERL	10.75	1 (2.1)
AI	6-21.5	4 (8.5)
Turner	14-20.5	2 (4.3)
PMDS	9.75	1 (2.1)
CAH	4-20.5	8 (17)
Other	3.75-8.5	9 (19.2)

AIS: androgen insensitivity syndrome; CAH: congenital adrenal hyperplasia; MGD: mixed gonadal dysgenesis; PAIS: Partial androgen insensitivity syndrome; PMDS: persistent mullerian duct syndrome;, Other: endocrine or anatomic syndromes, vanishing testes.

Sex assignment was concordant with genotype in 63.8% of cases at birth and in 76% of cases following GMT assessment (Table 2). Similarly, phenotype was concordant in 86.6% of cases at birth compared to 97.7% concordance after GMT assessment (Table 3). Due to small numbers, a trend in concordance of genotype and phenotype with sex assignment was seen, although statistical significance was not confirmed (Tables 2 and 3). Assessment of the individual features considered during the process of sex assignment demonstrated that concordance was higher following assessment by a GMT (Table 4).

**Table 2:** Concordance of genotype with sex assignment

		**Concordanceof karyotype with sex assignment after GMT assessment**	
		**Not concordant**	**Concordant**
**Concordance of karyotype with sex assignment at birth**	Not concordant	9 (19.1%)*	6 (12.8%)*
	Concordant	2 (4.3%)*	30 (63.8%)*

*McNemar p-value = 0.289.

**Table 3:** Concordance of phenotype with sex assignment

		**Concordanceof phenotype with sex assignment after GMT assessment**	
		**Not concordant**	**Concordant**
**Concordance of phenotype with sex assignment at birth**	Not concordant	0 (%)*	5 (11.4%)*
	Concordant	1 (2.3%)*	38 (86.4%)*

*McNemar p-value = 0.219.

**Table 4:** Factors assessed in determination of sex assignment

**Factors assessed at birth**	**Male sex assignment (%)**	**Female sex assignment (%)**	**Kappa agreement with factor**
Karyotype	46.8	53.2	0.371*
External anatomy	47.7	52.3	0.774*
Internal anatomy	42.9	57.1	0.571*
**Hormones	55.6	44.4	0.283
**Factors assessed during GMT evaluation**	**Male sex assignment (%)**	**Female sex assignment (%)**	**Kappa agreement with factor**
Karyotype	46.8	53.2	0.520*
External Anatomy	56.8	43.2	0.954*
Internal Anatomy	50.0	50.0	0.714*
**Hormones	63.0	37.0	0.557*

* Statistically significant at p < 0.05.

** Refers to hormonal testing to rule in or rule out presence of androgens. Normal female and male levels in children were considered the normal cut offs.

## Discussion

Managing patients with DSD involves complex medical and surgical challenges, as well as psychological and social uncertainties that cannot always be anticipated at the time of birth. This issue is not unique to this era of medical care, as earlier literature suggested that gender identity involved genetics, endocrinology, neurosurgery, psychology, and anthropology [10]. It has also been suggested that environmental agents (phytoestrogens) may plays a role in gender identity, in addition to genetic and hormonal influence [10]. Over the course of many decades, the management of patients with DSD has evolved to include a well-defined multidisciplinary team in the complex decision-making process of sex assignment [4,5]. An important component of our GMT assessment at TCH is the education and engagement of the parents in the decision-making process. We were one of the first teams to include parental education and involvement [4], which was a significant departure from the old paternalistic approach whereby the physician made the sex-assignment. The results of this study provide important data for taking the next step in establishing standardized management criteria. Our results suggest that our approach yields more satisfactory results than does the historical approach.

In spite of this and other advancements, the specific management is not clearly defined for each individual diagnosis, and the challenge of establishing guidelines for making decisions regarding sex assignment remains, partly due to the paucity of outcomes data in this population. Redefining the nomenclature used to refer to these patients in the context of their genetic diagnosis [5] has provided some clarity and was a step in the direction of standardization. Additionally, the benefits of a comprehensive evaluation that includes hormone, imaging, cytogenetic, and molecular studies are well documented, as is the recognition that a team approach is preferred. Finally, some patients may also benefit from diagnostic laparoscopy and gonadal biopsy to aid in the final sex determination when warranted. This series of interventions, as well as parental involvement, is critical toward establishing a standardized course of action that will eventually lead to a successful outcome for each patient.

The objective of this retrospective chart review was to compare the concordance of sex assignment as male or female with genotype or phenotype based on the initial presentation prior to GMT assessment and concordance following GMT assessment. Our chart review of existing patients demonstrates conclusively that sex assignment does not correlate exactly with phenotypic or genotypic features alone. The findings reported in this retrospective analysis are important preliminary steps toward ongoing studies to establish standardized management criteria.

## Conclusion

Decisions regarding sex of rearing in infants born with ambiguous genitalia are challenging, yet critical toward optimizing outcomes for these patients. To date, no data are available to establish guidelines regarding sex assignment, limiting the ability of a multidisciplinary team to diagnose and treat complex conditions effectively. The preliminary results of this study indicate that a greater correlation exists between phenotype and final sex assignment, which is important information for formulating standardized practice guidelines and decision-making algorithms in sex assignment among patients born with DSD. The limitations of our study include the retrospective design and small numbers lending to some interpretation bias. In addition, there were a variety of patients with DSD, therefore, the power within groups was limited. We plan to conduct further studies to ascertain the predictability of outcomes on the basis of sex assignment and underlying conditions. This patient population would benefit greatly from studies to assess patient satisfaction and the risks and benefits of specific gender assignments.

## Competing interests

The authors declare that they have o competing interests.

## Authors’ contributions

DS carried out the initial data collection on patients in the GMT clinic. JED and LPK participated in the design of the study. DP and JC participated in the initial draft and outline of the paper. OBS conducted all statistical analyses. JED and LPK participated in the final review and edits to the manuscript. All authors read and approved the final manuscript.
